# Investigation of the Effect of Process Parameters on Bone Grinding Performance Based on On-Line Measurement of Temperature and Force Sensors

**DOI:** 10.3390/s20113325

**Published:** 2020-06-11

**Authors:** Lihui Zhang, Lei Zou, Donghui Wen, Xudong Wang, Fanzhi Kong, Zhongyu Piao

**Affiliations:** 1 Key Laboratory of Special Purpose Equipment and Advanced Processing Technology, Zhejiang University of Technology, Ministry of Education & Zhejiang Province, Hangzhou 310014, China; lhzhang@zjut.edu.cn (L.Z.); Leizou_fan@126.com (L.Z.); franzkong@zjut.edu.cn (F.K.); piaozy@zjut.edu.cn (Z.P.); 2 Key Laboratory of Energy Thermal Conversion and Control of Ministry of Education, School of Energy and Environment, Southeast University, Nanjing 210096, China; xdwang_seu@seu.edu.cn

**Keywords:** bone grinding, temperature sensor, force sensor, orthogonal test method

## Abstract

This study investigates the effect of process parameters on neurosurgical bone grinding performance using a miniature surgical diamond wheel. Bone grinding is an important procedure in the expanded endonasal approach for removing the cranial bone and access to the skull base tumor via nasal corridor. Heat and force are generated during the grinding process, which may cause thermal and mechanical damage to the adjacent tissues. This study investigates the effect of grinding process parameters (including the depth of cut, feed rate, and spindle speed) on the bone grinding performance using temperature and force measurement sensors in order to optimize the grinding process. An orthogonal experimental design with a standard orthogonal array, L_9_ (3^3^), is selected with each parameter in three levels. The experimental results have been statistically analyzed using the range and variance analysis methods in order to determine the importance order of the process parameters. The results indicate that the effect of the cutting depth on the grinding temperature and normal force is the largest, while the effect of the spindle speed on the tangential force is the largest. A high spindle speed would make the temperature rise to a certain extent; however, it significantly reduces the grinding force. At a certain spindle speed, a lower depth of cut and feed rate help to reduce the grinding temperature and force.

## 1. Introduction

**Figure 1:**
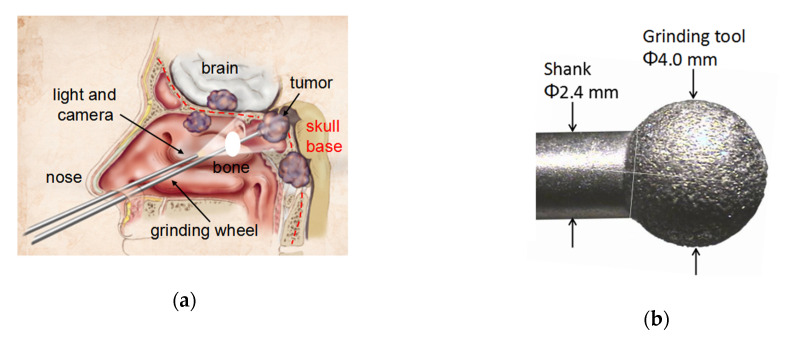
(**a**) The endonasal approach to the skull base. Reprinted from [1] with permission of Elsevier, and (**b**) spherical bone grinding tool. Reprinted from [2] with permission of Elsevier.

In recent years, the endoscopic endonasal approach, which is a minimally invasive skull base surgery procedure, has been widely applied in order to access the disease sites deep inside the brain. This approach uses the nostrils as natural corridors to directly access the tumor, without the need for large incisions [1], as shown in Figure 1a. The light source and camera enter through one nostril, and the grinding wheel passes through the other nostril, which is used to grind the bone for access to the disease site in the skull base for brain surgery. Therefore, it is less traumatic when compared with the open skull surgery in the skull base procedure. For brain surgery with an endoscopic endonasal approach, surgeons use a miniature ball-shape diamond grinding wheel, as shown in Figure 1b, in order to remove the bone, to expose and access the disease site in the skull base [1,2]. Usually, it also requires extensive grinding of the bone surrounding the optic, cavernous sinus, and branches of the trigeminal nerves, in order to identify and preserve these critical nerves during the resection of a brain tumor [3].

During the bone grinding process, the level of temperature rise and tool penetration force are two key factors that affect the outcome of surgery. Excessive heat and force generation could produce unnecessary thermal and mechanical damage to the bone and the surrounding nerves and vessels. Bones and nerves are especially vulnerable to high temperatures. It has been reported that bone grinding can generate a significant amount of heat, which leads to bone necrosis, thermal injury to the surrounding structures, and blood coagulation [4,5]. Overload grinding forces could potentially result in bone breakthrough and cracking [6,7].

The bone tissue is vulnerable to the elevated temperature. The threshold temperature for bone necrosis is 50 °C and even lower (43 °C) for nerves [2,3,8,9,10]. Pandey [10] reviewed the studies on the determination of threshold for thermal osteonecrosis. It was believed that an average temperature of 47 °C for 1 min. as threshold, above which the necrosis of the human bone will take place [11]. Many studies have been conducted in order to investigate the temperature rise in bone machining. The experimental method is based on direct temperature measurement by using thermocouples and thermagraph [12,13]. Recently, experimental methods coupled to the computational methods has been common practice to analyse the heat generation problem in bone machining. Fernandes [14,15] performed a thermal analysis in drilling using the three-dimensional dynamic finite element and experimental model.

The current status of bone grinding force and temperature research are reviewed. Tai et al. [16] presented a mechanistic model to predict the forces experienced during bone grinding, with application to haptic feedback for virtual reality surgical simulations. Grinding heat is of great concern; Shih et al. [2,17,18] developed a thermal model utilizing the inverse heat transfer method in order to predict the heat generation of bone grinding for skull base neurosurgery. The concept of cryogenic saline mist cooling [19] and nanoparticle jet mist cooling [20] were proposed separately, with their application in bone grinding, to suppress the grinding heat. Some scholars [21,22] have also mentioned that blockage might occur during bone grinding, which might increase the grinding temperature and force. In this case, new grinding wheels have been developed that are coated with submicron-sized titanium dioxide particles in order to generate hydrophobic surfaces, in the hope of preventing strong loads of bone swarf. The investigation into bone grinding is still not complete, and no research has been reported regarding the control of grinding heat and force from the perspective of the grinding processing parameters. Our work is to fill this gap.

Bone drilling and cutting are also widely applied in surgery, especially in trauma, dentistry, and plastic surgery. As is well known in the machining process, the process parameters have great impact on the process quality [23,24]. There are many publications of bone drilling and cutting focusing on the optimization of the process parameters [6,7,25,26,27,28,29,30,31,32]. For example, Singh et al. [6] investigated the optimization of drilling parameters, such as rotational speed, feed rate, and the type of tool, for the surface roughness and material removal rate using the Taguchi optimization method. Sui et al. [7] performed a complete analysis on the effect of the drilling process parameters, such as the drill geometry parameters and bone material type, on the drilling forces. Pandey et al. [25] presented a hybrid algorithm (Taguchi combined with membership function) for the optimization of the bone drilling process with multiple performance characteristics in order to minimize the drilling-induced bone tissue damage. Sezek et al. [26] investigated the influence of drill parameters on bone temperature and necrosis via FEM modelling and in vitro experiments. Li et al. [27] performed a simulation study on the effect of the cutting parameters and cooling mode on the bone-drilling temperature field while using brazed step and twist drills. Alam et al. [28] studied the level of forces produced during a chisel-like tool penetration in a fresh cortical bone. It was demonstrated that the depth of cut was the main factor affecting the level of cutting force for both of the conventional ultrasonically assisted cutting tools. Gupta et al. [29] studied the effect of various parameters on temperature distribution in conventional and diamond-coated hollow tool bone drilling. It was found that a lower spindle speed, feed rate, and drill diameter generated a lower temperature for both drill bits. From the review of the above literature, it can be seen the effect of the process parameters on the bone drilling or cutting performance was fully investigated. However, to the authors’ best knowledge, the effect of the process parameters on bone grinding has not been reported.

The objective of this study is to investigate the influence of the process parameters on the bone grinding temperature and force while using the orthogonal test method. Firstly, the experimental setup is introduced, followed by the experimental design. The standard orthogonal array, L_9_(3^3^), is adopted to design nine sets of tests under different process parameters—namely, the depth of cut, feed rate, and spindle speed. Subsequently, the range and variance analysis are utilized to perform the data analysis and to determine the influence trend and importance order of each factor on the grinding temperature and force.

## 2. Experimental Section

### 2.1. Experimental Setup

**Figure 2:**
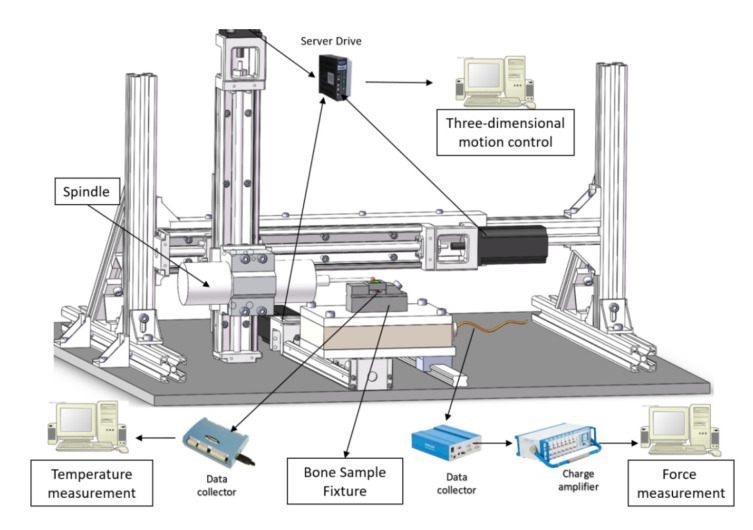
Schematic view of the three-dimensional bone grinding system.

The experimental setup is built to simulate the bone grinding operation during the surgical procedure. Figure 2 shows the overall system of the bone grinding experimental platform. It consists of four sub-systems, including a motion control subsystem, sample and spindle clamping subsystem, temperature measurement subsystem, and force measurement subsystem.

**Figure 3:**
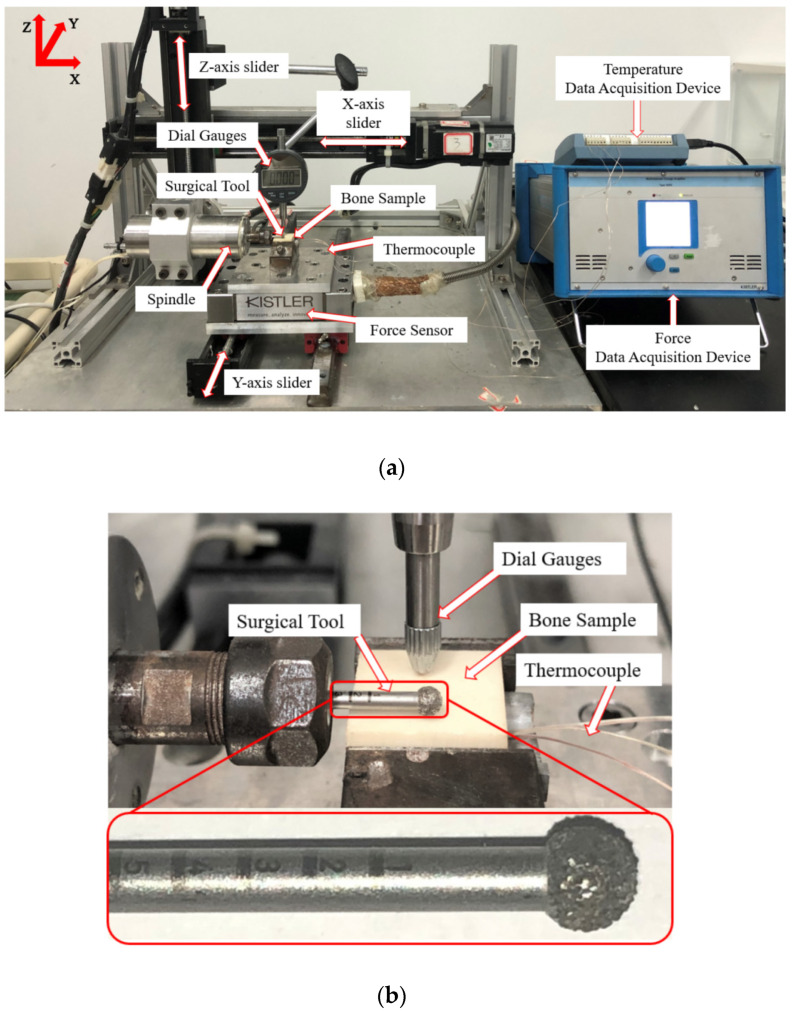
Photographs of the (**a**) overall experimental setup and a (**b**) close-view of the grinding wheel and workpiece.

Figure 3 shows the actual experimental setup. The motion control subsystem was built by three high-precision modules (HIWIN KK5002P-300A1-F2, Hangzhou, China) and a servo motor (HIWIN FRLS102B5A4C, Hangzhou, China), so as to control the linear motion of the grinding tools along the *X*, *Y*, and *Z* directions, in order to achieve high-precision positioning with a resolution of 20 μm. A spindle was horizontally fixed on the *Z*-direction module to follow the motion of a three-dimensional linear platform. A miniature spherical grinding tool (Stryker 4.0 mm round diamond bur Coarse, REF 5820–13–40, Kalamazoo, MI, USA) was attached to the spindle to remove the bone material. The grid size of the diamond tool was 711FEPA.

In surgery, the miniature ball-shaped diamond grinding wheel is usually held by hand by surgeons to remove the bone material in a paint brush motion. Therefore, in our study, the grinding wheel was set to feed along the *Y* direction, at a certain feed rate and depth of cut, so as to simulate the real bone grinding process. A dial indicator was applied to check the bone surface flatness after clamping in order to ensure a consistent depth of cut along the feed direction. The force sensor (model 9129A) was placed under the bone and it was connected to a data acquisition system to sample the forces in the *X*, *Y*, and *Z* directions simultaneously at 1 kHz.

**Figure 4:**
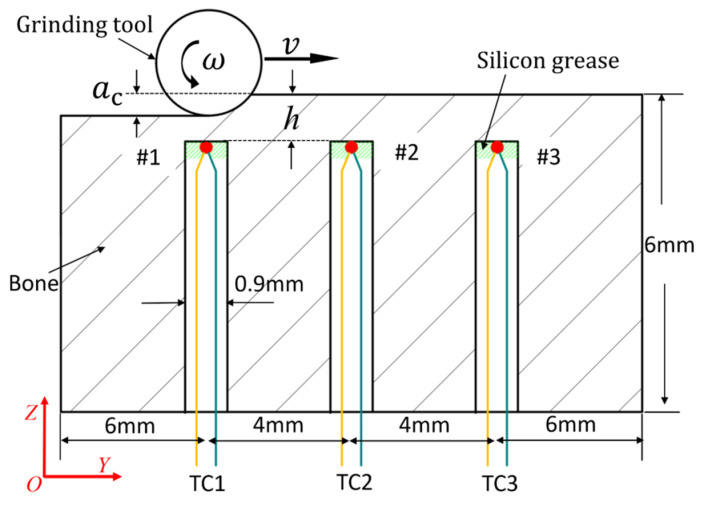
Schematic view of thermocouples’ arrangement.

Grinding-induced bone temperature rise was measured using temperature sensors of three K-type thermocouples. Figure 4 shows the thermocouples’ arrangement from a cross-section view along the center line of the ground groove. Three thermocouples (marked as TC1, TC2, and TC3) were dispersed along the grinding groove to observe the temperature changes and ensure the accuracy of temperature measurement results. The thermocouples were embedded at a depth of *h* underneath the bone surface through blind holes #1, #2, and #3. These blind holes were drilled from the back side of the bone sample using a 0.9 mm diameter drill. *h* is defined as the distance between the bone surface and the bottom of the blind hole. *a*_c_ is defined as the depth of cut. *h* is set at 100 μm, slightly larger than *a*_c_, so as to prevent the tips of the thermocouples being ground. Silicon grease was used on the tip of the thermocouples in order to reduce the thermal resistance of heat conduction. The thermocouples in the experiments (36 gauge K type, Omega Engineering, Shanghai, China) had a maximum variation of 1.1 °C according to the data sheet, which is acceptable in engineering.

### 2.2. Experiment Material

**Figure 5:**
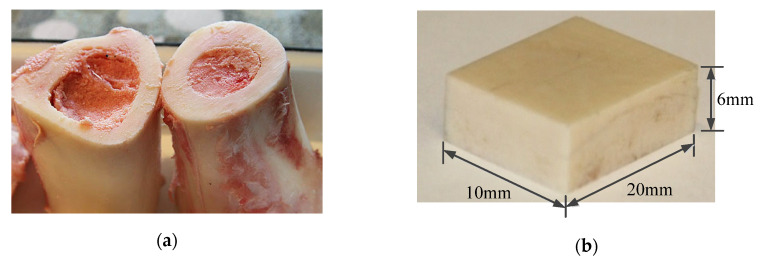
(**a**) Fresh bovine femur and (**b**) bone sample.

In our experiment, we used one kind of bone to make the sample, which is from the femur portion of fresh bovine bone, to minimize the influence of heterogeneous characteristics of bone tissue. It was obtained from a local slaughterhouse. No animals were sacrificed especially for the purpose of the current study. The bovine bone is most similar to human bones in terms of mechanical and thermal properties [6], so it was used as an experimental material in this study. Figure 5 shows the fresh bovine femur and the bone sample. The bone samples were processed, as follows: firstly, the original femur bone was cut into pieces with dimensions of 20 × 10 × 6 mm^3^. Subsequently, the bone samples were placed on a high-precision milling machine to finish the surface, so as to make their roughness lower than Ra 0.6 μm.

### 2.3. Design of Experiments

**Figure 6:**
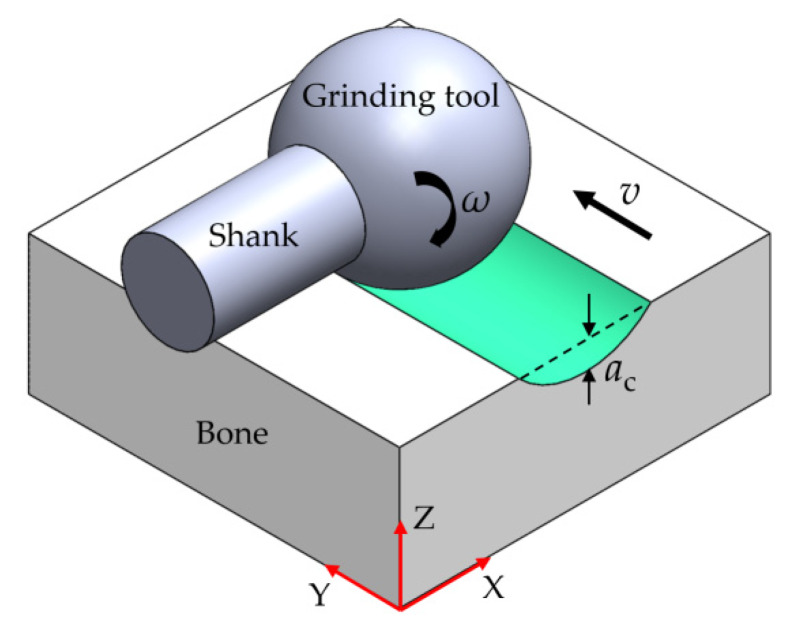
Schematic view of bone grinding process.

Figure 6 shows the overall configuration of the shank, spherical grinding tool, and bone. The grinding tool, with a rotational speed (*ω*), moves forward along a + *Y* direction at a depth of cut (*a*_c_). In our study, three grinding process parameters, the depth of cut (*a*_c_), feed rate (*v*), and spindle speed (*ω*), were considered as the variables. An orthogonal experimental design [33,34] was selected in order to find out the importance order of these parameters. The orthogonal test in this study was designed with the help of a standard orthogonal array L_9_(3^3^), as shown in Table 1. Depth of cut (Factor A), feed rate (Factor B), and spindle speed (Factor C) were chosen as the main factors to be considered. Each factor had three levels, which were determined based on the knowledge of previous studies [16,17,22]. In total, there were nine possible combinations. It should be noted that interactions between two factors were not considered.

## 3. Experimental Results

**Figure 7:**
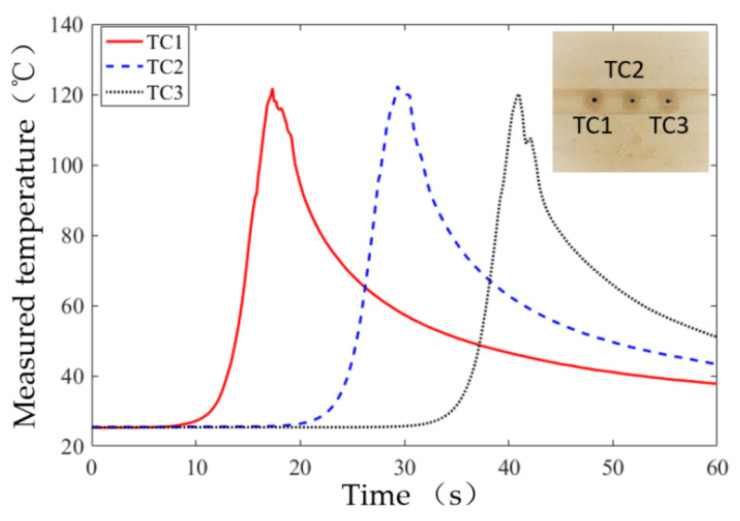
Temperature measurements in the bone grinding experiment using temperature sensors of three thermocouples (marked as TC1, TC2, and TC3).

**Figure 8:**
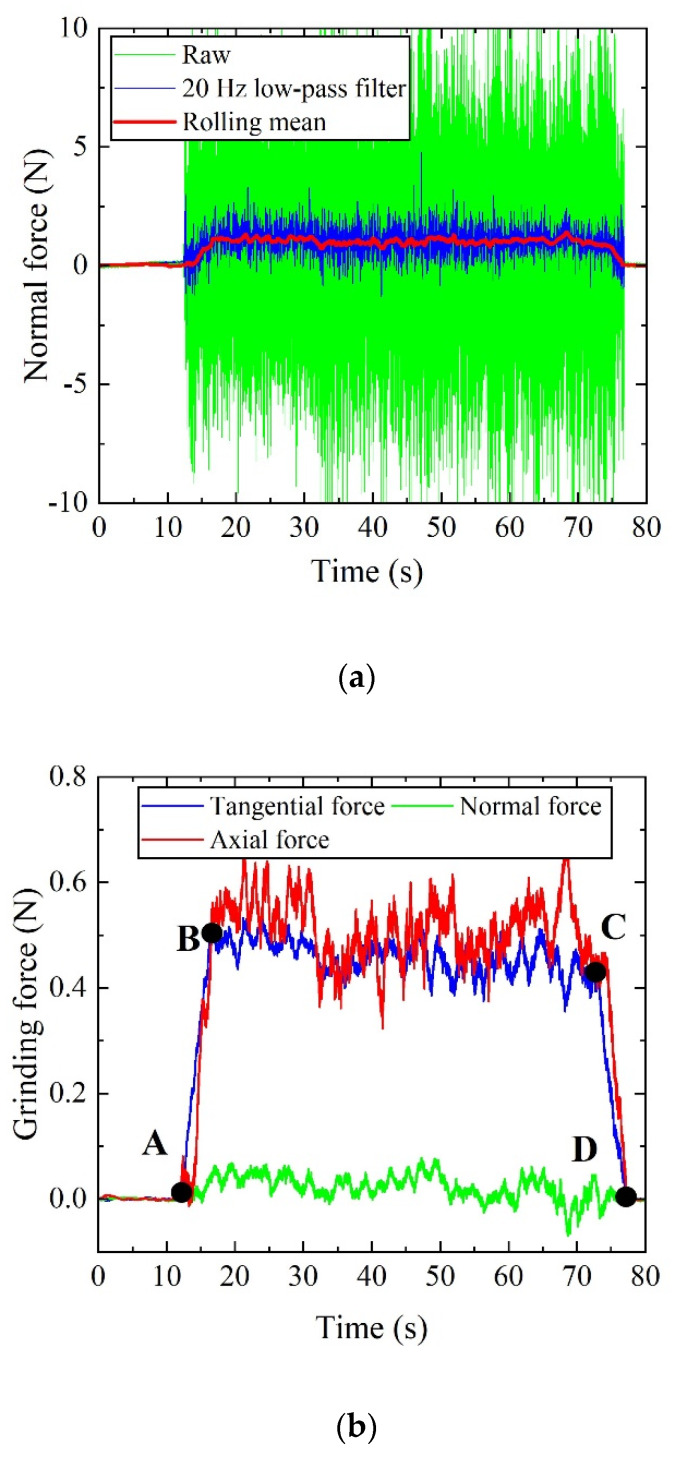
(**a**) Force profile recorded during an experiment. (**b**) Profiles of tangential force, axial force and normal force after filtering and smoothing.

Figure 7 and Figure 8, respectively, show the typical profiles of the temperature and force measurement data. They were measured under the condition of a cutting depth of 0.4 mm, feed rate of 20 mm/min, and spindle speed of 20,000 rpm. In Figure 7, the three temperature sensors of the thermocouples, TC1, TC2, and TC3, had consistent temperature results, which indicate that the temperature measurement is reliable. As TC1-TC3 were placed 100 μm directly below the bone–wheel interface, the peak value of the measured temperature could approximate the maximum temperature of bone grinding. In this case, the average of the peak temperatures of TC1-TC3 was taken as the maximum grinding temperature for the subsequent data analysis in Section 4.

Figure 8 shows the force data that were recorded during bone grinding experiment. In Figure 8a, the raw data is highly oscillatory partly due to the vibrations generated by the motor coupled with the system resonance. What’s more, the abrasive burrs on the grinding wheel have many small cutting edges, and each of which generates an oscillatory force. The average of the oscillatory force profile represents the resistance due to grinding on the bone workpiece. A low-pass filter was applied to eliminate the effect of noise and then generate the rolling mean in order to find the mean resistance force, as shown by the red line in Figure 8a. The dynamometer comes with a software of DynoWare, which has the function of low-pass filtering and smoothing (rolling mean) available for use. The cut-off frequency was set as 20 Hz, which can eliminate the effect of random errors (in general, higher than 50 Hz), and meanwhile reflect the variation characteristics of grinding force. Figure 8b shows the profiles of tangential force, axial force, and normal force after data filtering and smoothing. It is observed the magnitudes of tangential and normal forces are equivalent, while the axial force is very small and can be neglected. The marked points of A and D represent the moments when the grinding tool cut-in and cut-out of the workpiece, respectively. The region between B and C is regarded as the stable grinding stage, so the force data in this region are used to calculate the mean force.

By using the above data processing method, Table 2 summarizes the experimental results of maximum grinding temperature (denoted as *T*_max_), tangential force (denoted as *F_t_*), and normal force (denoted as *F_N_*) of all nine tests. With various parameters at different levels designed by orthogonal test method, the measured maximum temperature varies from 65 °C to 119 °C, the tangential force from 0.17 N to 0.53 N, and the normal force from 0.268 N to 0.503 N. It should be noted that the maximum temperature, which exceeded the threshold value of 50 °C, was measured under dry grinding conditions. In real clinical procedures, saline irrigation is typically used to flood the bone-wheel interface in order to reduce heat generation. The amount of coolant should not be too large for the small space of the surgical site, otherwise it will affect the vision of the endoscopic surgery. Therefore, the effect of flood irrigation on reducing the temperature increase might be limited. In this manuscript, we focused on investigating the influence of different process parameters on the grinding temperature and force in order to optimize the process parameters, so as to minimize thermal and mechanical damage during bone grinding. The ranges of process parameters were selected based on previous studies [16,17,22], so as to reasonably emulate a clinical condition. With the optimized set of parameters and the effect of the coolant, the grinding temperature and force could be controlled in a safer range.

## 4. Statistical Analysis and Discussion

The purpose of the data analysis was to find the effect trend of various factors on the experimental results, and which factor had a significant impact on the grinding temperature and force. In the following text, the range and variance analysis methods were adopted in order to perform the statistical analysis for the experimental results.

### 4.1. Range Analysis

The range analysis is a statistical method for calculating the factors’ sensitivity to the experimental results that were obtained from the orthogonal experiment. The calculation process of range analysis is shown in Equations (1)–(3), according to the literature [35], as follows:

(1) $$K_{\mathrm{Xm}}={\bar{I}}_{\mathrm{Xm}}$$

(2) $$G_{0X}=\max\left(K_{X1},\;K_{X2},\;K_{X3}\right);\;G_{1X}=\min\left(K_{X1},\;K_{X2},\;K_{X3}\right)$$

(3) $$R_{X}=G_{0X}-G_{1X}$$

In Equation (1), ${\bar{I}}_{\mathrm{Xm}}$ stands for the average value of the experimental results, which contain the factor X (X = A, B, C) with level *m* (*m* = 1, 2, 3). Taking the first level of factor A, for example, ${\bar{I}}_{A1}=(y_{1}+y_{2}+y_{3})/3$, here, *y_i_* represents the experimental result (*T*_max_, *F_t_* or *F_N_*) of test *i*. In Equation (2), *G*_0X_ and *G*_1X_ are defined as the maximum and minimum values of *K*_Xm_. *R*_X_ in Equation (3) is defined as the range, which is the difference between the extreme values of data *K*_Xm_. *R*_X_ stands for the influence degree of factor X. The greater the range, the more sensitive the factor.

According to the experimental results presented in Table 2, the average values, K*_Xm_,* and the influence degree, *R*_X_, can be calculated using Equations (1)–(3), as shown in Table 3. With regard to the influence of the factors on the grinding temperature, the following was found, *R*_A_ (32.7) > *R*_B_ (17.3) > *R*_C_ (16.3). The difference between *R*_X_ indicates the order of impact. Therefore, the effect of the depth of cut (Factor A) was the largest, followed by the feed rate (Factor B) and the spindle speed (Factor C). The values of *R*_B_ (17.3) and *R*_C_ (16.3) were very close, which indicated an equivalent influence degree.

With regard to the tangential force, the effect of the spindle speed (Factor C) was the most significant. The order of impact was *R*_C_ (0.263) > *R*_A_ (0.151) > *R*_B_ (0.087), as observed from Table 3. It indicates that the feed rate (Factor B) has a relatively smaller effect on the tangential force, *F_t_*.

However, the order of impact with regard to the normal force is a bit different from the tangential force, which is given by *R*_A_ (0.164) > *R*_C_ (0.120) > *R*_B_ (0.080). This shows that the effect of the depth of cut (Factor A) was the greatest, while the effect of the feed rate (Factor B) was the smallest.

### 4.2. The Effect of Process Parameters on Grinding Temperature

**Figure 9:**
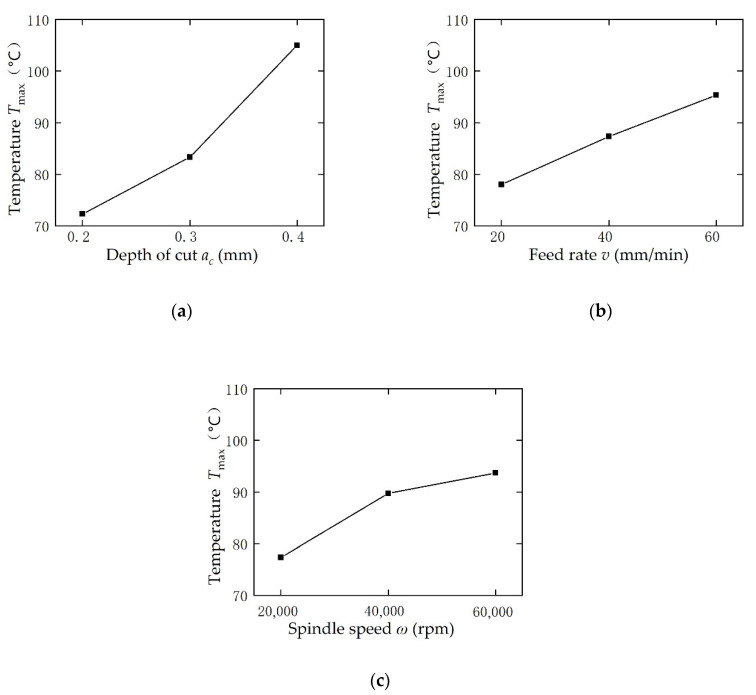
Variation of the bone grinding temperature with a variation of process parameters.

The effect of depth of cut (Factor A), feed rate (Factor B), and spindle speed (Factor C) on grinding temperature was visualized in Figure 9 by using the data of *K*_A*m*_, *K*_B*m*_ and *K*_C*m*_ (*m* = 1,2,3) in Table 3. From Figure 9a, it was observed that the grinding temperature (*T*_max_) significantly increases with an increase in the cutting depth (*a*_c_). When *a*_c_ rises from 0.2 mm to 0.4 mm, the *T*_max_ has increased by 32.7 °C. Moreover, the slope of temperature rise curve tends to increase with an increase of *a*_c_, which is due us using a spherical grinding tool, in this case, although the depth of cut is increased by the same amount, the volume of material removal is increased more than double. Figure 9b shows the effect of feed rate (*v*). When *v* increased from 20 to 60 (mm/min), the grinding temperature rises by 17.3 °C in an approximately linear way. Larger spindle speed will make the grinding temperature rise, as seen from Figure 9c. In summary, smaller depth of cut, feed rate, and spindle speed are beneficial for lowering down the grinding temperature.

The phenomenon observed in Figure 9 is reasonable according to the grinding theory. That is because larger depth of cut and feed rate would make the material removal per unit time increase and consume more mechanical energy; as a result, more heat is produced. A larger spindle speed can reduce the maximum chip thickness of a single abrasive; therefore, increasing the specific energy of grinding, which results in more heat generation and high temperature.

### 4.3. The Effect of Process Parameters on Grinding Force

**Figure 10:**
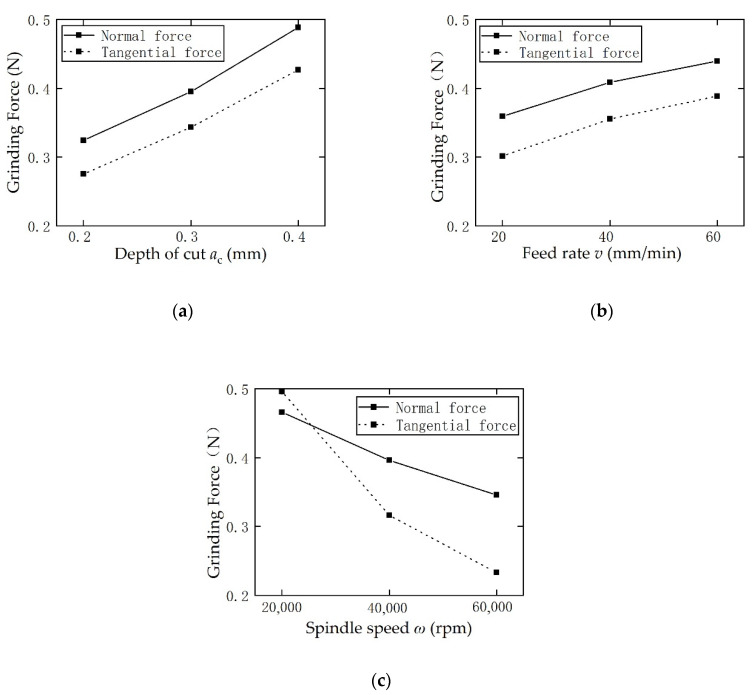
Variation of bone grinding force with variation of process parameters.

Figure 10 illustrates the effect of the depth of cut (Factor A), feed rate (Factor B), and spindle speed (Factor C) on the grinding force. When the depth of cut (*a*_c_) increases, the tangential force (*F_t_*) and normal force (*F_N_*) both rise up with a quasi-linear profile. It was found that *F_t_* and *F_N_* increased by 0.15~0.16 N when the *a*_c_ varied from 0.20 to 0.40 mm. The feed rate had a similar influence trend to that of the cutting depth with a smaller increase of 0.08 N. However, the tangential force decreased by 0.26 N and the normal force reduced by 0.12 N with the spindle speed varying from 20,000 to 60,000 rpm. Especially for the tangential force, it declined up to 53%. This is because, when the spindle speed is high, the chip thickness of a single abrasive grain is reduced, and the abrasives are more easily to cut into the bone. Therefore, a high spindle speed is recommended for controlling the bone grinding force.

### 4.4. Variance Analysis

The analysis of variance (ANOVA) was performed to further verify the credibility of the conclusions obtained from the range analysis method, and the results are shown in Table 4.

The magnitude of *F*_X_ (*X* = A, B, C) reflects the level of significance. With a given significance level (*α*), the corresponding critical *F*_1-_*_α_* value can be found by referring to the *F* distribution table. For example, given *α* = 0.05*,* *F*_0.95_(2,2) = 19*, α* = 0.01*,* and *F*_0.99_(2,2) = 99. The significance level of factor *X* was characterized by comparing the *F*_X_ value with the *F*_1-_*_α_* value. In general, if *F*_0.95_(2,2) < *F_X_* *≤* *F*_0.99_(2,2), the effect of factor *X* is regarded as being significant. If *F_X_* *>* *F*_0.99_(2,2), the effect of factor *X* is considered to be more significant. The results of the variance analysis show that the three discussed process parameters are all important for the grinding temperature and force. The factor that has the greatest influence on the temperature and axial force is the depth of cut (Factor A), while the factor that has the greatest influence on the tangential force is the spindle speed (Factor C). The effect of the feed rate (Factor B) on the experimental results is relatively smaller. The order of importance is A > B > C for the grinding temperature, C > A > B for tangential force, and A > C > B for the normal force. This is consistent with the conclusions that were obtained by the range analysis.

## 5. Conclusions

In this study, an experimental study was conducted in order to investigate the effect of process parameters on grinding performance based on the orthogonal test method. A *L*_9_(3^3^) orthogonal table was designed with the help of three factors and three levels, and the orthogonal test results included the grinding maximum temperature (*T*_max_), tangential force (*F_t_*), and normal force (*F_N_*). The range analysis and variance analysis are applied to compare the influence degree of each factor on the test results. The analysis results help to draw the following conclusions: (1) the effect of the cutting depth (Factor A) on the grinding temperature is the largest, and the effect of feed rate (Factor B) and spindle speed (Factor C) are much smaller and equivalent. For the grinding force, both the cutting depth (Factor A) and spindle speed (Factor C) have a significant impact, and the feed rate has little effect; and, (2) with the increase of the cutting depth, feed rate, and spindle speed, the maximum grinding temperature increases. However, a high spindle speed helps to effectively reduce the grinding force; and (3), while taking into account the time of surgery, it is recommended to use a high spindle speed, small depth of cut, and proper feed rate.

In the endonasal endoscopic surgery, the bone grinding process might be affected by the fluid interaction, including the blood and saline solution. The impact of saline solution (coolant) should be greater, due to it being dropped continuously to the grinding site for cooling in order to prevent the potential thermal injury. Therefore, in the future, we need to integrate the effect of saline irrigation to our bone grinding experimental model.

This well-developed method can be used to characterize and compare the bone grinding performance under different grinding process parameters. The bone grinding temperature and force will also be affected by the abrasive size, feed direction, and contact angle etc., which still need further investigation. The method proposed in this article is also suitable for analyzing the influence of the above factors, and it will be our future work.

## Figures and Tables

**Table 1:** Grinding process parameters and their considered levels.

Factors	Units	Level 1	Level 2	Level 3
Depth of cut (A)	*a*_c_ (mm)	0.2	0.3	0.4
Feed rate (B)	*v* (mm/min)	20	40	60
Spindle speed (C)	*ω* (rpm)	20,000	40,000	60,000

**Table 2:** L_9_ (3^3^) Orthogonal array and experimental results.

Test No.	Factors			Experimental Results		
	A	B	C	*T*_max_ (°C)	*F_t_* (N)	*F_N_* (N)
	*a*_c_ (mm)	*v* (mm/min)	*ω* (rpm)			
1	1 (0.20)	1 (20)	2 (40,000)	65	0.202	0.282
2	1 (0.20)	2 (40)	3 (60,000)	81	0.170	0.268
3	1 (0.20)	3 (60)	1 (20,000)	71	0.454	0.423
4	2 (0.30)	1 (20)	3 (60,000)	81	0.172	0.293
5	2 (0.30)	2 (40)	1 (20,000)	73	0.504	0.472
6	2 (0.30)	3 (60)	2 (40,000)	96	0.354	0.420
7	3 (0.40)	1 (20)	1 (20,000)	88	0.530	0.503
8	3 (0.40)	2 (40)	2 (40,000)	108	0.392	0.486
9	3 (0.40)	3 (60)	3 (60,000)	119	0.358	0.476

**Table 3:** Range analysis of orthogonal test results.

Index	Level		Factors	
		A	B	C
*T*_max_ (°C)	1	*K*_A1_ = 72.3	*K*_B1_ = 78.0	*K*_C1_ = 77.3
	2	*K*_A2_ = 83.3	*K*_B2_ = 87.3	*K*_C2_ = 89.7
	3	*K*_A3_ = 105.0	*K*_B3_ = 95.3	*K*_C3_ = 93.7
	*R_X_*	32.7	17.3	16.3
*F_t_* (N)	1	*K*_A1_ = 0.275	*K*_B1_ = 0.301	*K*_C1_ = 0.496
	2	*K*_A2_ = 0.343	*K*_B2_ = 0.355	*K*_C2_ = 0.316
	3	*K*_A3_ = 0.427	*K*_B3_ = 0.389	*K*_C3_ = 0.233
	*R_X_*	0.151	0.087	0.263
*F_N_*(N)	1	*K*_A1_ = 0.324	*K*_B1_ = 0.359	*K*_C1_ = 0.466
	2	*K*_A2_ = 0.395	*K*_B2_ = 0.409	*K*_C2_ = 0.396
	3	*K*_A3_ = 0.488	*K*_B3_ = 0.440	*K*_C3_ = 0.346
	*R_X_*	0.164	0.080	0.120

**Table 4:** Variance analysis of measured grinding temperature, tangential force, and normal force.

Index	*F*_A_	*F*_B_	*F*_C_	*F*_critical_		Significant Factors
				(α = 0.05)	(α = 0.01)	
Temperature (*T*_max_)	116.33	32.33	32.04	19	99	A
Tangential force (*F_t_*)	88.33	29.87	277.35	19	99	C
Normal force (*F_N_*)	136.38	33.30	72.24	19	99	A

A-depth of cut; B-feed rate; C-spindle speed.
